# 2-Hy­droxy­pyridinium *p*-toluene­sulfonate

**DOI:** 10.1107/S1600536812019873

**Published:** 2012-05-12

**Authors:** Yu Jin

**Affiliations:** a Ordered Matter Science Research Center, Southeast University, Nanjing 211189, People’s Republic of China

## Abstract

In the title molecular salt, C_5_H_6_NO^+^·C_7_H_7_O_3_S^−^, the cations and anions are connected by N—H⋯O and O—H⋯O hydrogen bonds, forming [100] chains.

## Related literature

For general background on ferroelectric frameworks, see: Zhang *et al.* (2008 ▶, 2009 ▶, 2010 ▶). For related salts containing *p*-toluene­sulfonate anions, see: Helvenston *et al.* (2006 ▶); Collier *et al.* (2006 ▶); Koshima *et al.* (2001 ▶).
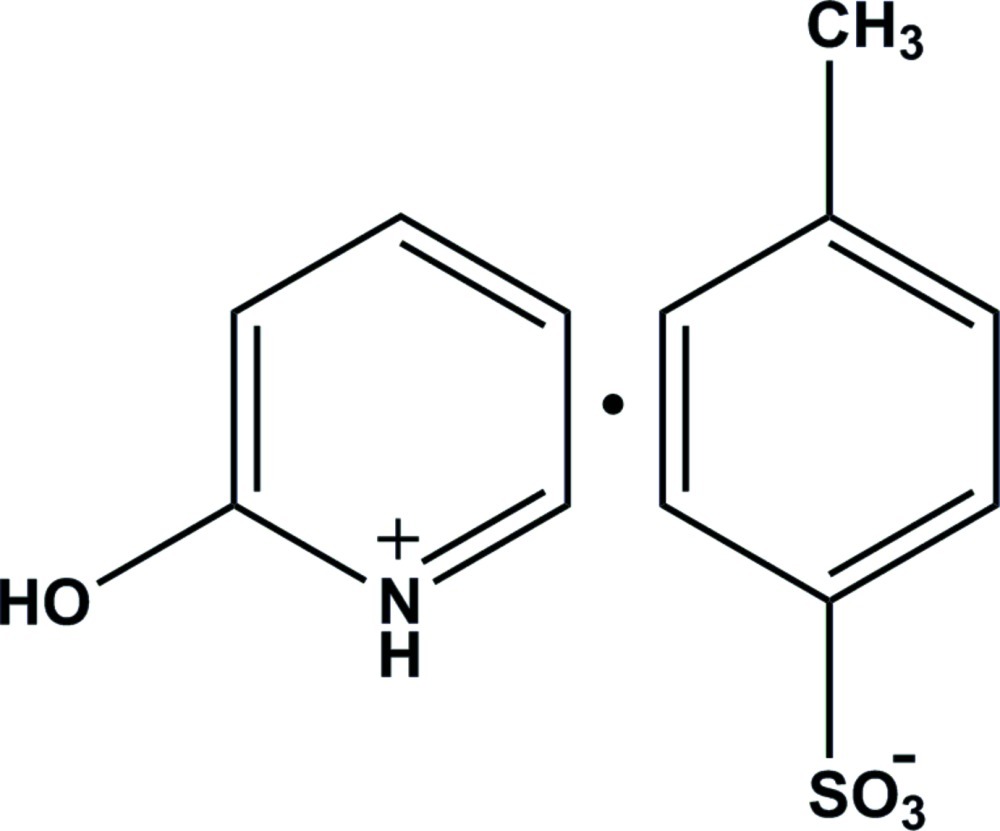


## Experimental

### 

#### Crystal data

C_5_H_6_NO^+^·C_7_H_7_O_3_S^−^
*M* *_r_* = 267.29
Orthorhombic, 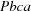

*a* = 10.293 (2) Å
*b* = 14.484 (3) Å
*c* = 15.708 (3) Å
*V* = 2341.8 (8) Å^3^
*Z* = 8
Mo *K*α radiation
μ = 0.28 mm^−1^
*T* = 293 K
0.3 × 0.3 × 0.2 mm

#### Data collection

Rigaku Mercury CCD diffractometer
Absorption correction: multi-scan (*CrystalClear*; Rigaku, 2005 ▶) *T*_min_ = 0.489, *T*_max_ = 1.000
23178 measured reflections
2688 independent reflections
2239 reflections with *I* > 2σ(*I*)
*R*_int_ = 0.075

#### Refinement

*R*[*F* ^2^ > 2σ(*F* ^2^)] = 0.046
*wR*(*F* ^2^) = 0.111
*S* = 1.09
2688 reflections
169 parameters
H atoms treated by a mixture of independent and constrained refinement
Δρ_max_ = 0.26 e Å^−3^
Δρ_min_ = −0.41 e Å^−3^

### 

Data collection: *CrystalClear* (Rigaku, 2005 ▶); cell refinement: *CrystalClear*; data reduction: *CrystalClear*; program(s) used to solve structure: *SHELXS97* (Sheldrick, 2008 ▶); program(s) used to refine structure: *SHELXL97* (Sheldrick, 2008 ▶); molecular graphics: *SHELXTL* (Sheldrick, 2008 ▶); software used to prepare material for publication: *SHELXL97*.

## Supplementary Material

Crystal structure: contains datablock(s) I, global. DOI: 10.1107/S1600536812019873/bh2431sup1.cif

Structure factors: contains datablock(s) I. DOI: 10.1107/S1600536812019873/bh2431Isup2.hkl

Supplementary material file. DOI: 10.1107/S1600536812019873/bh2431Isup3.cml

Additional supplementary materials:  crystallographic information; 3D view; checkCIF report

## Figures and Tables

**Table 1:** Hydrogen-bond geometry (Å, °)

*D*—H⋯*A*	*D*—H	H⋯*A*	*D*⋯*A*	*D*—H⋯*A*
O1—H1⋯O2	0.89 (4)	1.66 (4)	2.523 (2)	160 (3)
N1—H1*D*⋯O4^i^	0.86	1.86	2.704 (2)	166

Symmetry code: (i) 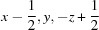.

**Fig. 1.**
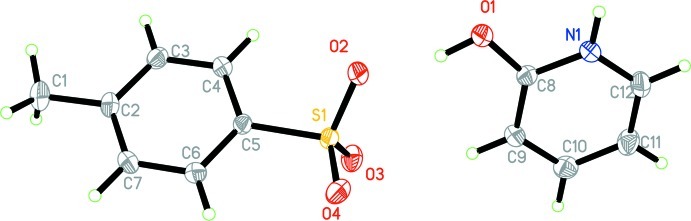
The structure of the title compound with displacement ellipsoids drawn at the 30% probability level.

**Fig. 2.**
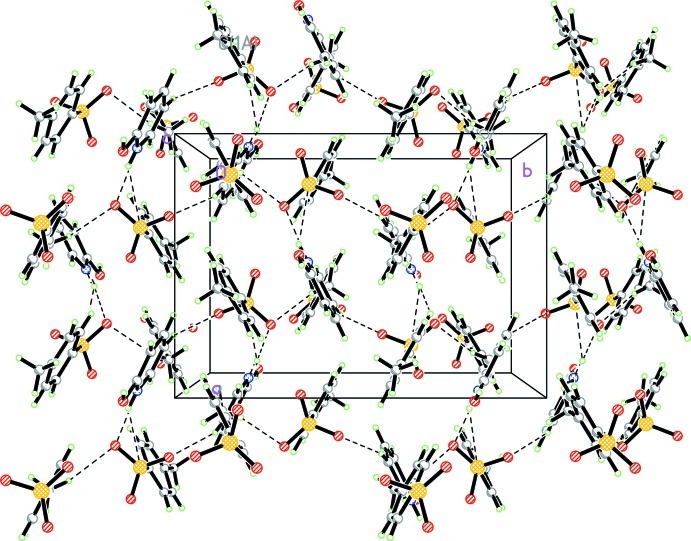
Part of the crystal structure of the title compound viewed along the c axis. Intermolecular interactions are shown as dashed lines.

## supplementary crystallographic information

### Comment

Several crystal structures of *p*-toluenesulfonate have been reported previously (Helvenston *et al.*, 2006; Collier *et al.*, 2006; Koshima *et al.*, 2001). As an extension of this research, we report here the synthesis and the crystal structure of the title complex.

Until now, researchers have found that molecular motion can cause a rotation of the local structure to give rise to the formation of reversible structural phase transition from high-temperature disordered fashion to low temperature ordered fashion. Reversible structural phase transition is caused by many kinds of factors. Hydrogen-bonding interactions come to be the most common factor. Transition for hydrogen-bonding interactions from high-temperature disordered state to low temperature ordered state allows reversible structural phase transition. The compound reported here could have a tendency to hold such transition as a result of numerous hydrogen-bonding interactions in its crystal structure. The transition from the disordered arrangement to the ordered one gives rise to sharp change in the physical properties of these compounds. Only few compounds in which the components can be arranged in a disordered status at a relative high temperature and in an ordered one at a relative low temperature have been found until now (Zhang *et al.*, 2008, 2009, 2010). As part of our search for simple ferroelectric compounds we have investigated the title compound and report its room temperature structure.

The asymmetric unit, containing one anion and one cation, is shown in Fig. 1 with the hydrogen bonds listed in Table 1. The compound remains stable as a result of the existence of numerous hydrogen-bonding interactions formed in the crystal. These interactions tie the cations and anions together in a complex spatial geometry (Fig. 2).

### Experimental

C_5_H_6_ON^+^.C_7_H_7_O_3_S^-^ was formed from a mixture of C_5_H_5_ON (95.1 mg, 1.00 mmol), C_7_H_7_SO_3_H (172 mg, 1.00 mmol), and distilled water (5 mL), which was stirred a few minutes at room temperature, giving a clear transparent solution. After evaporation for a few days, block colourless crystals suitable for X-ray diffraction were obtained in about 71% yield and filtered and washed with distilled water.

### Refinement

H atoms bound to C and N atoms were placed at idealized positions [C—H = 0.93–0.96 Å, N—H = 0.86 Å] and allowed to ride on their parent atoms with *U*_iso_ = 1.5 *U*_eq_(C1) for the methyl group and *U*_iso_ = 1.2 *U*_eq_(carrier atom) otherwise. Hydroxyl H atom was found in a difference map and refined freely.

### Crystal data

**Table:**

C_5_H_6_NO^+^·C_7_H_7_O_3_S^−^	*F*(000) = 1120
*M**_r_* = 267.29	*D*_x_ = 1.516 Mg m^−^^3^
Orthorhombic, *P**b**c**a*	Mo *K*α radiation, λ = 0.71073 Å
Hall symbol: -P 2ac 2ab	Cell parameters from 3450 reflections
*a* = 10.293 (2) Å	θ = 6.2–55.3°
*b* = 14.484 (3) Å	µ = 0.28 mm^−^^1^
*c* = 15.708 (3) Å	*T* = 293 K
*V* = 2341.8 (8) Å^3^	Block, colourless
*Z* = 8	0.3 × 0.3 × 0.2 mm

### Data collection

**Table:**

Rigaku Mercury CCD diffractometer	2688 independent reflections
Radiation source: fine-focus sealed tube	2239 reflections with *I* > 2σ(*I*)
Graphite monochromator	*R*_int_ = 0.075
ω scans	θ_max_ = 27.5°, θ_min_ = 3.1°
Absorption correction: multi-scan (*CrystalClear*; Rigaku, 2005)	*h* = −13→13
*T*_min_ = 0.489, *T*_max_ = 1.000	*k* = −18→18
23178 measured reflections	*l* = −20→20

### Refinement

**Table:**

Refinement on *F*^2^	Secondary atom site location: difference Fourier map
Least-squares matrix: full	Hydrogen site location: inferred from neighbouring sites
*R*[*F*^2^ > 2σ(*F*^2^)] = 0.046	H atoms treated by a mixture of independent and constrained refinement
*wR*(*F*^2^) = 0.111	*w* = 1/[σ^2^(*F*_o_^2^) + (0.0362*P*)^2^ + 1.2062*P*] where *P* = (*F*_o_^2^ + 2*F*_c_^2^)/3
*S* = 1.09	(Δ/σ)_max_ < 0.001
2688 reflections	Δρ_max_ = 0.26 e Å^−^^3^
169 parameters	Δρ_min_ = −0.41 e Å^−^^3^
0 restraints	Extinction correction: *SHELXL97* (Sheldrick, 2008), Fc^*^=kFc[1+0.001xFc^2^λ^3^/sin(2θ)]^-1/4^
0 constraints	Extinction coefficient: 0.0347 (16)
Primary atom site location: structure-invariant direct methods	

### Fractional atomic coordinates and isotropic or equivalent isotropic displacement parameters (Å^2^)

**Table:**

	*x*	*y*	*z*	*U*_iso_*/*U*_eq_	
C1	0.5889 (2)	0.09901 (16)	0.81599 (13)	0.0461 (6)	
H1A	0.6382	0.0453	0.8315	0.069*	
H1B	0.4989	0.0890	0.8291	0.069*	
H1C	0.6203	0.1514	0.8473	0.069*	
C2	0.6034 (2)	0.11654 (13)	0.72297 (12)	0.0322 (4)	
C3	0.5108 (2)	0.08731 (14)	0.66618 (13)	0.0356 (5)	
H3	0.4365	0.0580	0.6862	0.043*	
C4	0.52611 (19)	0.10060 (14)	0.58087 (12)	0.0335 (5)	
H4	0.4625	0.0804	0.5431	0.040*	
C5	0.63523 (18)	0.14370 (13)	0.55080 (12)	0.0277 (4)	
C6	0.72725 (19)	0.17508 (14)	0.60638 (12)	0.0318 (4)	
H6	0.8007	0.2055	0.5863	0.038*	
C7	0.7102 (2)	0.16122 (13)	0.69220 (13)	0.0342 (5)	
H7	0.7728	0.1827	0.7301	0.041*	
C8	0.53344 (18)	0.14460 (13)	0.19333 (12)	0.0309 (4)	
C9	0.65239 (19)	0.10354 (14)	0.20266 (13)	0.0352 (5)	
H9	0.6890	0.0961	0.2564	0.042*	
C10	0.7158 (2)	0.07393 (14)	0.13220 (14)	0.0401 (5)	
H10	0.7970	0.0464	0.1378	0.048*	
C11	0.6624 (2)	0.08387 (17)	0.05305 (14)	0.0446 (5)	
H11	0.7052	0.0622	0.0049	0.054*	
C12	0.5470 (2)	0.12556 (16)	0.04656 (14)	0.0424 (5)	
H12	0.5097	0.1342	−0.0068	0.051*	
N1	0.48537 (16)	0.15481 (12)	0.11587 (10)	0.0344 (4)	
H1D	0.4112	0.1814	0.1101	0.041*	
O1	0.45846 (14)	0.17554 (12)	0.25338 (10)	0.0446 (4)	
H1	0.498 (3)	0.167 (2)	0.303 (2)	0.095 (12)*	
O2	0.53381 (14)	0.17679 (12)	0.40618 (9)	0.0477 (4)	
O3	0.70545 (15)	0.06576 (10)	0.41196 (9)	0.0426 (4)	
O4	0.75191 (14)	0.22574 (10)	0.42828 (8)	0.0397 (4)	
S1	0.65917 (5)	0.15323 (3)	0.44102 (3)	0.03132 (18)	

### Atomic displacement parameters (Å^2^)

**Table:**

	*U*^11^	*U*^22^	*U*^33^	*U*^12^	*U*^13^	*U*^23^
C1	0.0660 (16)	0.0449 (13)	0.0273 (11)	−0.0031 (11)	0.0006 (10)	−0.0002 (9)
C2	0.0433 (11)	0.0285 (10)	0.0247 (9)	0.0050 (8)	−0.0003 (8)	−0.0014 (7)
C3	0.0343 (11)	0.0397 (11)	0.0326 (10)	−0.0038 (9)	0.0056 (9)	−0.0006 (9)
C4	0.0306 (10)	0.0415 (11)	0.0286 (10)	−0.0020 (8)	−0.0021 (8)	−0.0025 (9)
C5	0.0277 (9)	0.0317 (10)	0.0238 (9)	0.0053 (7)	0.0003 (7)	−0.0008 (7)
C6	0.0293 (10)	0.0340 (10)	0.0322 (10)	−0.0016 (8)	0.0004 (8)	0.0006 (8)
C7	0.0358 (11)	0.0358 (10)	0.0310 (10)	−0.0011 (8)	−0.0075 (8)	−0.0037 (8)
C8	0.0290 (10)	0.0348 (10)	0.0290 (10)	−0.0036 (8)	0.0013 (8)	0.0003 (8)
C9	0.0335 (11)	0.0398 (11)	0.0321 (10)	0.0004 (8)	−0.0045 (8)	0.0026 (9)
C10	0.0370 (11)	0.0381 (11)	0.0451 (12)	0.0086 (9)	0.0020 (9)	0.0002 (10)
C11	0.0507 (13)	0.0492 (13)	0.0338 (11)	0.0092 (11)	0.0069 (10)	−0.0043 (10)
C12	0.0469 (13)	0.0534 (13)	0.0269 (10)	0.0036 (10)	−0.0019 (9)	−0.0005 (10)
N1	0.0289 (8)	0.0432 (10)	0.0309 (9)	0.0013 (7)	−0.0033 (7)	0.0022 (7)
O1	0.0335 (8)	0.0702 (11)	0.0302 (8)	0.0100 (7)	0.0000 (6)	−0.0018 (8)
O2	0.0349 (8)	0.0804 (12)	0.0276 (7)	0.0126 (8)	−0.0024 (6)	0.0046 (8)
O3	0.0529 (9)	0.0417 (9)	0.0331 (8)	0.0064 (7)	0.0033 (7)	−0.0075 (6)
O4	0.0386 (8)	0.0428 (8)	0.0377 (8)	0.0030 (6)	0.0099 (6)	0.0051 (7)
S1	0.0296 (3)	0.0402 (3)	0.0241 (3)	0.0057 (2)	0.00163 (18)	0.0007 (2)

### Geometric parameters (Å, º)

**Table:**

C1—C2	1.491 (3)	C8—N1	1.322 (2)
C1—H1A	0.9600	C8—C9	1.369 (3)
C1—H1B	0.9600	C9—C10	1.355 (3)
C1—H1C	0.9600	C9—H9	0.9300
C2—C7	1.364 (3)	C10—C11	1.367 (3)
C2—C3	1.373 (3)	C10—H10	0.9300
C3—C4	1.363 (3)	C11—C12	1.336 (3)
C3—H3	0.9300	C11—H11	0.9300
C4—C5	1.369 (3)	C12—N1	1.330 (3)
C4—H4	0.9300	C12—H12	0.9300
C5—C6	1.366 (3)	N1—H1D	0.8600
C5—S1	1.7474 (19)	O1—H1	0.89 (4)
C6—C7	1.374 (3)	O2—S1	1.4425 (15)
C6—H6	0.9300	O3—S1	1.4284 (15)
C7—H7	0.9300	O4—S1	1.4333 (16)
C8—O1	1.299 (2)		
			
C2—C1—H1A	109.5	O1—C8—C9	127.14 (19)
C2—C1—H1B	109.5	N1—C8—C9	118.81 (18)
H1A—C1—H1B	109.5	C10—C9—C8	118.75 (19)
C2—C1—H1C	109.5	C10—C9—H9	120.6
H1A—C1—H1C	109.5	C8—C9—H9	120.6
H1B—C1—H1C	109.5	C9—C10—C11	121.1 (2)
C7—C2—C3	118.42 (18)	C9—C10—H10	119.5
C7—C2—C1	120.61 (19)	C11—C10—H10	119.5
C3—C2—C1	120.97 (19)	C12—C11—C10	118.3 (2)
C4—C3—C2	120.99 (19)	C12—C11—H11	120.8
C4—C3—H3	119.5	C10—C11—H11	120.8
C2—C3—H3	119.5	N1—C12—C11	120.4 (2)
C3—C4—C5	119.90 (18)	N1—C12—H12	119.8
C3—C4—H4	120.0	C11—C12—H12	119.8
C5—C4—H4	120.0	C8—N1—C12	122.64 (18)
C6—C5—C4	120.00 (18)	C8—N1—H1D	118.7
C6—C5—S1	120.44 (15)	C12—N1—H1D	118.7
C4—C5—S1	119.49 (15)	C8—O1—H1	109 (2)
C5—C6—C7	119.34 (19)	O3—S1—O4	112.53 (9)
C5—C6—H6	120.3	O3—S1—O2	112.76 (10)
C7—C6—H6	120.3	O4—S1—O2	111.68 (10)
C2—C7—C6	121.31 (18)	O3—S1—C5	107.00 (9)
C2—C7—H7	119.3	O4—S1—C5	106.83 (9)
C6—C7—H7	119.3	O2—S1—C5	105.48 (9)
O1—C8—N1	114.05 (18)		

### Hydrogen-bond geometry (Å, º)

**Table:**

*D*—H···*A*	*D*—H	H···*A*	*D*···*A*	*D*—H···*A*
O1—H1···O2	0.89 (4)	1.66 (4)	2.523 (2)	160 (3)
N1—H1*D*···O4^i^	0.86	1.86	2.704 (2)	166
O1—H1···S1	0.89 (4)	2.73 (4)	3.6139 (18)	169 (3)
N1—H1*D*···S1^i^	0.86	2.75	3.4747 (18)	143

Symmetry code: (i) *x*−1/2, *y*, −*z*+1/2.
